# Histological and transcriptome analysis provides new insights into the hematopoietic and immune functions of head kidney, trunk kidney and spleen of adult large yellow croaker, *Larimichthys crocea*

**DOI:** 10.1016/j.cirep.2025.200223

**Published:** 2025-04-12

**Authors:** Aihua Zhong, Yingbin Wang, Haiqi Zhang, Xiaojun Yan, Shuo Jia

**Affiliations:** a College of Fishery, Zhejiang Ocean University, Zhoushan, Zhejiang 316022, China; b Zhejiang Institute of Freshwater Fisheries, Huzhou, Zhejiang 313001, China; c National Engineering Research Center of Marine Facilities Aquaculture, Marine Science and Technology College, Zhejiang Ocean University, Zhoushan, Zhejiang 316022, China

**Keywords:** *Larimichthys crocea*, Hematopoiesis, Immune function

## Abstract

Many teleosts possess a unique immune system because hematopoiesis occurs in multiple organs, such as the head kidney and trunk kidney. The head kidney (HK) and trunk kidney (TK) are considered immune organs in that they contain macrophages and melanomacrophage centers (MMCs). The large yellow croaker (*Larimichthys crocea*), a vital marine-cultured fish in Asia, lacks bone marrow and lymph nodes, relying instead on the head kidney (HK), trunk kidney (TK), and spleen for hematopoiesis and immune functions. This study provides a comprehensive histological and transcriptomic analysis of these organs in adult large yellow croaker, revealing their normal physiological roles in hematopoiesis and immunity. Histological examination identified hematopoietic tissues, lymphoid tissues, and melanomacrophage centers (MMCs) in all three organs, with the spleen exhibiting a higher abundance of MMCs. The MMCs and associated lymphocyte aggregates were observed in three organs, indicating the presence of the germinal center–like structures. Transcriptome analysis revealed organ-specific gene expression patterns, with 47, 1368 and 1529 genes only expressed in HK, TK and spleen, respectively. The key hematopoietic genes such as *runx1, cmyb, epor*, and *csf3r* were highly expressed in both HK and TK, indicating their primary roles in hematopoiesis. The spleen, a major lymphoid organ, also expressed the hematopoietic genes. Immune-related genes, including *tlr3, tlr7*, and *tlr8*, were highly expressed in the spleen, highlighting its role in antiviral responses. Additionally, complement genes such as *c6* and *c7* were highly expressed in HK and TK, while *c8γ* was uniquely expressed in TK. These findings enhance our understanding of the molecular mechanisms underlying hematopoiesis and immune defense in teleosts, providing valuable insights for disease management in aquaculture.

## Highlights

• The key hematopoiesis-related genes were identified in three organs.
• C8γ was only observed in trunk kidney.
• The MMCs and associated lymphocyte aggregates were also observed in three organs.

## Introduction

Teleosts, as lower vertebrates, have a unique immune system compared to higher vertebrates, because they lack bone marrow and lymph nodes [1]. Bone marrow, a distinctive class of tissue that fills the cores of larger bones in humans and other animals, is principally responsible for the sustained blood cell production in adult mammals [2,3]. Lymph nodes, oval-shaped structures in humans, serve as efficient sites for immune surveillance due to their sophisticated microarchitectures [4]. They can filter the foreign matters from blood, produce antibody by activation of B cells and control bacteria diseases through blood–lymph–blood circulation network [5]. Despite lacking bone marrow and lymph nodes, teleosts possess a similar immune architecture that incorporates both innate and adaptive immune responses, such as phagocytosis of various types of phagocytes, the antibody production by B cells and cellular immunity by T cells, enabling them to effectively combat a diverse range of pathogens [6,7].

Where and how the blood cells are produced have been a subject of intense study. Catton (1951) conducted comprehensive surveys of hematopoietic tissue in specific teleosts and found that in common roach (*rutilus*), the kidney serves as the hematopoietic organ, while in perch (*Perca fluviatilis*), it is the spleen. In trout (*Salmo trutta*), both the kidney and spleen function as hematopoietic organ, based on histological evidence [8]. Subsequent research has confirmed that the kidney and spleen have hematopoietic functions in many teleost species, which can support the differentiation and maturation of the various blood cells [[8], [9], [10], [11]].

The kidney of many teleosts is composed of two distinct parts: head kidney (HK) and trunk kidney (TK) [9,[12], [13], [14]]. The HK lacks nephrons and has no renal function after the embryo hatching. The parenchyma of HK is the immune-related tissue, supported by the kidney stroma. The stroma is a reticulo-endothelial stroma composed of endothelial cells, the reticular cells and other cells [15]. The TK, located along the ventral side of the vertebral column, contains renal and hematopoietic tissues [14,16,17]. Similar to mammals, TK of fish contains nephrons with a glomerulus, renal tubules (proximal and distal tubules), and collecting ducts. The hematopoietic cells are present in renal interstitium [18]. Furthermore, the kidney can function as a secondary immune organ, largely exerted by B cells, T cells and macrophages [19,20]. The lymphocytes in kidney tissue are observed by morphological approaches [17]. The B cell and T cell population in HK have been characterized using single cell transcriptional approaches [[21], [22], [23]]. The macrophages can coalesce to form macrophage aggregates, also known as melanomacrophage centers (MMCs) [17,24]. These macrophages and MMCs are essential for responding to exogenous materials, including infectious pathogens [25]. Additionally, MMCs contribute to antigen recognition [[26], [27], [28]]. Dendritic cells, as antigen-presenting cells (APCs), can process and present protein antigens to specific T-cells, and have been identified in HK and TK in Salmon, rainbow trout, and grass carp [17,29,30]. Thus, HK and TK are complex and unusual organs that house distinct functional systems, including immune and hematopoietic systems and others.

The spleen, as an important secondary lymphoid organ, plays a crucial role in both innate and adaptive immune responses. It contains lymphoid tissue, MMCs and granulosa cells [15]. In addition, the spleen performs the hematopoietic function in some fish species [8,10,31]. The presence of hematopoietic stem/progenitor cells (HSPCs) in zebrafish spleen has been demonstrated at single cell resolution [32].

Although functioning in hematopoiesis and immune defense, the similarities and differences among three organs are not fully understood. In many fish, HK is considered the primary organ of definitive hematopoiesis [9,10,33]. During development, the genes of the immunoglobulin heavy chain begin to express at pro-B or pre-B1 cell stage in mice [34]. Interestingly, several important genes are only expressed in yellow catfish TK, such as the genes encoding the immunoglobulin light and heavy chain, indicating TK plays an important role in B cell development [35]. HK-derived neutrophils do not release DNA after stimulation with recombinant CXCL8s and CXCb1, while TK-derived cells can release neutrophil extracellular traps after CXCb1 stimulation [36]. This phenomenon may be attributed to differences in the maturation state of neutrophils, implicating TK plays a role in regulating neutrophil maturation. The resting B cells, activated B cells and Ig-secreting plasma cells are observed in HK, TK and spleen of rainbow trout; however, the main populations of B cell subsets are not same, more plasma cells presenting in HK, more resting B cells in spleen, more active B cells in TK [20]. After *Vibrio anguillarum* infection, the differential expressed genes (DEGs) are enriched in Ubiquitin-mediated proteolysis pathway in red sea bream HK; whereas, DEGs are enriched in intestinal immune network for IgM production pathway in the spleen [37]. The similar phenomena are observed in yellow catfish after *Aeromonas hydrophila* stimulation, with 1139 and 1117 genes significantly changed in TK and HK, correspondingly. The upregulating level of IgM in TK is higher than that in HK after yellow catfish are infected by *Edwardsiella ictaluri*, indicating more IgM^+^ cells are present in TK [35]. Together, the functions of three organs needs further exploration during hematopoiesis and immune defense.

Aquaculture has developed rapidly recently due to overfishing and the increased human population. Large yellow croaker (*Larimichthys crocea*) are the vital marine cultured fish in China and other Asian countries. The China Fishery Yearbook shows that the yield of large yellow croaker was the highest among mariculture fish in 2023. It has attracted substantial attention due to its delicious taste and high nutritional content. Recent studies have focused on its farming technologies, disease prevention, genetics, and physiological functions [[38], [39], [40], [41], [42]]. Many studies have been conducted to understand the immune mechanisms of the large yellow croaker, particularly focusing on molecular characterization and disease resistance applications. Many immune-related genes and proteins have been identified, such as *tlr21*, gal-1, *cspg4*, Rip2, with high expression levels in immune organs like the spleen, highlighting their roles in pathogen recognition and antiviral responses [[43], [44], [45], [46]].

However, the detailed mechanisms of hematopoiesis and immune defense in this species, especially the roles of HK, TK, and spleen, have not been fully elucidated. Transcriptome data of different organs can provide deeper insights into what constitutes a specific organ, how organ normally functions, and how changes of gene activity may reflect disease [47]. By combining histological and transcriptome analyses, we provide a comprehensive profile of gene expression and cellular organization in these organs. The identification of organ-specific and differentially expressed genes, particularly those related to immune and hematopoietic functions, offers valuable insights into the molecular mechanisms underlying their roles. The results not only enhance our knowledge of the immune system of large yellow croaker but also have practical implications for disease management and welfare in aquaculture.

## Materials and methods

### Ethics statement and sample collection

Large yellow croaker collection and experiments were performed following the Administration Regulations on Laboratory Animals of China and were approved by the Committee on Laboratory Animal Welfare and Ethics of Zhejiang Ocean University.

Large yellow croaker was obtained from the breeding base near Zhejiang Ocean University, China. Six specimens (weight 300 ± 50 g, four females and two males, 30-month-old) were anesthetized, and their HK, TK, and spleen were collected for subsequent experiments. The tissues from two individuals were pooled together to generate a mixed specimen, and each tissue for RNA sequencing contained three biological replicates.

### Histological examination

The HK, TK and spleen were collected to prepare cell smears and paraffin-embedded tissue sections. Blood for smears was withdrawn from the branchial vein with sodium citrate (3.8 %) as an anticoagulant. The blood smears, tissues-imprint slides and tissue sections were prepared using the previously reported methodology [35]. The smears and sections were investigated under an Olympus CX23 microscope. The various cell types were identified according to Fijan's terminology [48].

### RNA isolation

Total RNA was extracted from tissues using an RNA purification reagent (Invitrogen). The genomic DNA was eliminated using the DNase I RNase-free kit (TaKaRa). The concentration of RNA was measured by ND-2000 (NanoDrop Technologies), and the quality was verified by the bioanalyzer 2100 (Agilent). RNA with high-quality standards (RNA integrity number > 9, 260/280 > 1.8) was retained for subsequent experiments.

### Construction of cDNA library and sequencing

cDNA sequencing libraries were generated using the TruSeqTM RNA sample preparation Kit (Illumina, San Diego, CA). Firstly, 2 μg total RNA was used to purify messenger RNA through the poly A adsorption using oligo (dT) magnetic beads. Subsequently, enriched mRNA was fragmented into approximately 200 bp fragments by fragmentation buffer. Following the instructions of the SuperScript double-stranded cDNA synthesis kit (Invitrogen, CA), double-stranded cDNA was synthesized by random hexamer primers. After end repair and adding single A bases and adaptors, the double-stranded cDNA was amplified with 15 PCR cycles. Then, cDNA sequences were purified using electrophoresis (2 % Low Range Ultra Agarose) and library quality was assessed using TBS380. Finally, the purified products were sequenced using the Illumina HiSeq NovaSeq 6000, and paired-end reads were obtained (read length: 2 × 150 bp).

### Read mapping and transcriptome assembly

The adapters and Poly-A stretches were trimmed, and the low-quality reads were filtered with the default parameters of FASTP (https://github.com/OpenGene/fastp) software [49]. Subsequently, the high-quality (quality score > 30 bp) reads were independently mapped to the large yellow croaker reference genome (GCF_000972845.2) using the HISAT2 package [50]. The mapped clean reads were assembled into contigs with a reference-based method using StringTie2.

### Functional annotation

After assembly, functional annotation was performed by aligning the contigs against six databases, including NCBI-NR, Pfam (Protein family), KEGG (Kyoto Encyclopedia of Genes and Genomes), GO (Gene Ontology), Swiss-Prot and KOG/COG (Clusters of Orthologous Groups of proteins) databases. The TransDecoder software was used to predict the coding sequence region (CDS) (https://github.com/TransDecoder/TransDecoder, v5.5.0). The transcription factor was analyzed using Animal Transcription Factor Database AnimalTFDB 4.0.

### Analysis of differentially expressed genes (DEGs) and enrichment analysis

The gene abundance, as indicated by the number of transcripts per kilobase of exon model per million mapped reads (TPM), was calculated using RSEM. The lowly-expressed genes, with fewer than three reads, were filtered. To analyze gene expression differences between three tissues, the fold change (FC) and adjusted P value (Padj) of each gene were calculated using the DEseq2 packages. The genes with |log2 FC| > 1 and Padj  < 0.05 were selected as DEGs for further analysis. The Padj value represents the Benjamini–Hochberg FDR-corrected p-value. The DEGs were then annotated in the GO and KEGG databases using Goatools and KOBAS to identify the enriched GO terms and KEGG pathways.

### Quantitative real-time PCR

Fluorescence quantitative real-time PCR was conducted as previously mentioned. First, reverse transcription was performed using iScript Q RT SuperMix for qPCR (with gDNA wiper) (Vazyme, China) with random hexamers. Expression level analyses were conducted using the ChamQ SYBR Color qPCR Master Mix kit (Vazyme Biotech Co., Ltd, China). The PCR reactions were conducted as follows: 5 min at 95 °C, 5 s at 95 °C, 30 s at 55 °C, and 40 s at 72 °C, repeating 40 cycles in the last 3 steps. All qRT-PCR reactions were performed in triplicate. The reference gene was β-actin. Primer 5 software was used to design the primers (Table 1).

**Table 1:** Primers of candidate genes for qRT-PCR.

Gene description	NCBI Accession Number	Primer sequence (5′ to 3′)
Colony stimulating factor 3 receptor (*Csf3r*)	LOC104919598	Forward: TCGTAGGAGGCGTGGACATCAG Reverse: GGGTGTTAGGAGTGGTGAGGTTTG
Complement 7 (*C7*)	LOC104920675	Forward: CGGATGGATGTGGAGACAGATTTCG Reverse: AGTGTCACAAGTTGCGGGAAGTTC
Integrin subunit alpha 3 (*Itga3*)	LOC104923253	Forward: AACGGCAGATTGTGGTGGATGATG Reverse: CGATGACTGAGCAGCGTGATGG
Toll-like receptor 5 (*Tlr5*)	LOC 104,927,236	Forward: GTGAGGACAGTATGTGGACGCTTG Reverse: AGGCAGAGCAGGAACCCAGTAG
β-actin	LOC104932075	Forward: GACCTGACAGACTACCTCATG Reverse: AGTTGAAGGTGGTCTCGTGGA

## Result

### Histological structure of HK, TK, and spleen

**Fig. 1:**
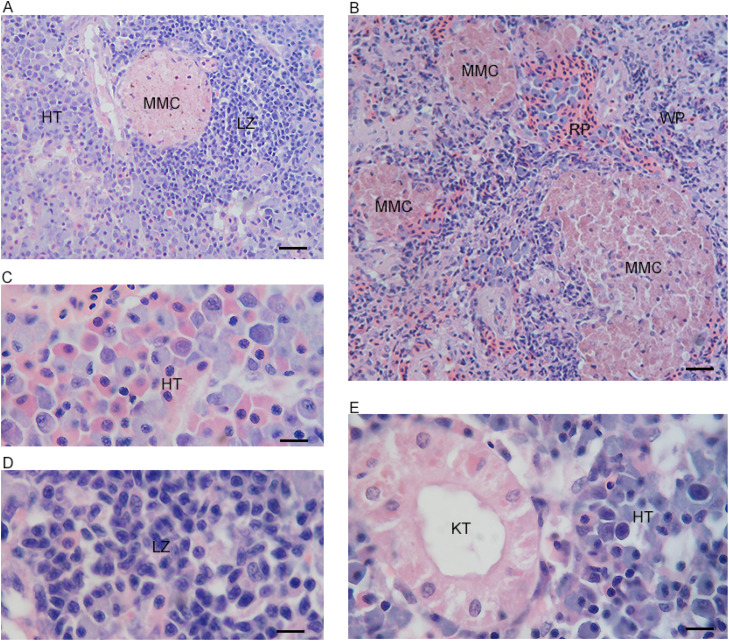
The section from the large yellow croaker. (A) The section from the head kidney (10 × 40). (B) The section from the spleen (10 × 40). (C) The hematopoietic tissue (HT) in the head kidney (10 × 100). (D) The lymphoid zone in the spleen (10 × 100). (E) The section from the trunk kidney (10 × 100). Melanomacrophage aggregate. (MMC). White pulp (WP). Red pulp (RP). Lymphoid zone (LZ). Kidney tubule (KT).

The HK and TK of the large yellow croaker were located ventrally along the dorsal column, from the base of the skull to the caudal region. The HK consisted of two lobes, right and left lobes. The cortex and the medulla were difficult to distinguish in the HK. The parenchyma of HK was composed of hematopoietic tissue, lymphoid tissue, and MMCs, but lacked nephrons tubules and renal capsules. MMCs were rounded, oval, or irregular outline (Fig. 1A, C, Supplementary Fig. 1A).

**Fig. 2:**
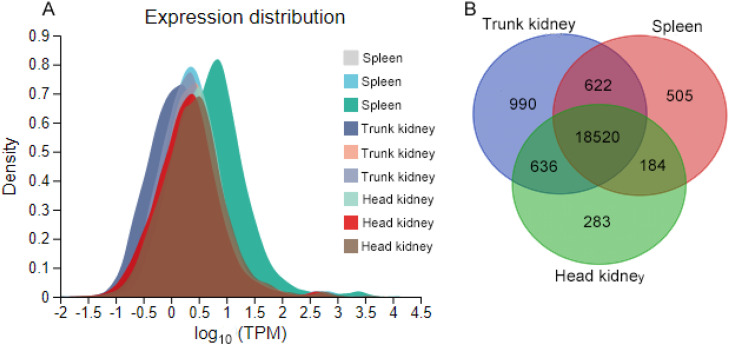
The expressed genes in three organs. (A) TPM density distribution with the x-axis being log_10_(TPM) and the y-axis being the density of log10(TPM). (B) The Venn diagram of expressed genes in three organs.

It was easy to distinguish the medulla from the cortex in TK due to their different histological structure. The cortex of TK contained hematopoietic tissue, MMCs, and lymphoid tissue. The medulla of TK mainly contained excretory components but also had hematopoietic tissue, lymphoid tissue, and MMCs. The lymphoid zone in HK and TK consisted of small-and medium-sized lymphocytes and a small number of other cell types, such as erythrocytes and monocytes (Fig. 1E, Supplementary Fig. 1B-C, Fig. 2A).

The spleen exhibited a well-defined architecture comprising white pulp and red pulp. The white pulp, constituting the majority of splenic tissue, was filled with hemopoietic cells, erythrocytes, and diverse immune populations—including granulocytes, lymphocytes, monocytes, and macrophages. The lymphocyte center or aggregation (small and medium-sized lymphocyte aggregation) were prominently detected within the white pulp. On the other hand, the red pulp was filled with erythrocytes and a limited number of macrophages. Many MMCs with a rounded, oval, or irregular outline were observed in the white pulp. The spleen had a higher abundance of MMCs than HK and TK, and larger MMCs were also observed in the spleen (Fig. 1B, Supplementary Fig. 1D, Figs. 2B-C, 1D). MMCs in the three organs contained heterogeneous or granular pigmented materials ranging from yellow to dark brown (Fig. 1A, Supplementary Fig. 2D). Notably, lymphocyte center or aggregation was observed in proximity to some MMCs (Fig. 1A, Supplementary Fig. 2B).

### Overview of sequence data

Two paired-end reads were generated on the Illumina platform, producing over 46 million raw reads. After filtering and data quality control, more than 46,422,226 clean reads with average lengths of 150 base pairs were obtained. The Q20 (percentage of nucleotides with a Phred quality score ≥ 20) and Q30 scores were greater than 97.63 % and 93.35 %, respectively. The GC (GC base) content of the read sequences exceeded 50 %, and the error rate (error rate determined through Qphred = −10 log_10_(e)) was less than 0.03 % (Table S1). Overall, more than 89.6 % of clean reads were mapped to the large yellow croaker genome. The clean reads were assembled into 59,576 transcripts using StringTie, with a maximum transcript length of 95,731 bp. The total number of assembled nucleotides was 204,906,663, with an average size of 3439 bp and an N50 length of 4666 bp. According to the length distribution, 51,846 (87.02 %) transcripts exceeded 1 kb, generally indicating a high annotation rate (Supplementary Fig. 3).

### Gene functional annotation

To understand the functions of the gene, the assembled transcripts were queried against KEGG, NR, Pfam, Swiss-Prot, COG, and GO databases. The analysis identified 24,432 annotated genes, including 17,202 genes in GO, 18,263 genes in KEGG, 22,707 genes in COG, 24,395 genes in NR, 21,547 genes in Swiss-Prot and 21,189 genes in Pfam. In addition, 12,784 genes (49.55 %) were annotated in all six public databases (Supplementary Fig. 5). The remaining transcripts had no significant match.

### Gene expression analysis

The gene expression was calculated by mapping obtained reads to the assembled transcripts using Bowtie software. The mapped reads were then converted into transcripts per kilobase of exon model per million mapped reads (TPM) with RSEM analysis. To better understand the function of the three tissues, we analyzed the gene expression profile, which resulted in the differences among tissues. The comparison of gene expression abundances in three tissues was presented as a TPM distribution diagram. In general, there were slight differences in the three tissues based on the TPM distribution diagram (Fig. 2A). The number of expressed genes varied among different organs. The largest number of expressed genes was detected in TK (20,768 annotated genes, gene counter ≥ 3), followed by the spleen (19,831 annotated genes) and HK (19,623 genes). Some expressed genes were tissue-specific, representing 1.44 % (HK) to 4.76 % (TK) of the total number of expressed genes in a given tissue (Fig. 2B). The TK had higher gene-specificity, with some immune-related genes observed only in this tissue, including *mast/stem cell growth factor receptor kita, activin A receptor type 1C* and *complement C8 gamma chain* (TPM:13.42). The *complement C8 alpha and beta* chains were only observed in the spleen, and *immunoglobulin superfamily member 10* (transcript variant X2) was only observed in the HK. In addition, 18,520 annotated genes were observed in three tissues, representing fundamental cellular processes (e.g., metabolism, cytoskeleton organization) and baseline hematopoietic/immune functions across organs.

### Organ-dependent differentially expressed genes (DEGs) analysis

**Fig. 3:**
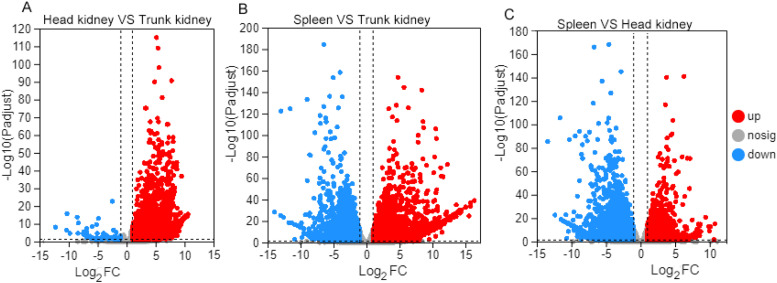
Differentially expressed genes (DEGs) among three organs. (A) Volcano plot of the DEGs between head kidney and trunk kidney. (B) Volcano plot of the DEGs between spleen and trunk kidney. (C) Volcano plot of the DEGs between spleen and head kidney.

The differentially expressed genes (DEGs) between any two tissues were identified using the R DEseq2. Interestingly, the number of DEGs varied depending on the pair of tissues compared. There were 1987 genes differentially expressed (Padj < 0.05, fold change (FC) > 2 or < 0.5) between the HK and TK, including 1925 highly expressed genes in TK and 62 highly expressed genes in the HK (Fig. 3A).

There were 4519 genes differentially expressed between HK and spleen, including 1672 highly expressed genes in HK and 2847 highly expressed genes in the spleen (Fig. 3C). Meanwhile, the comparison between spleen and TK revealed the highest number of DEGs, consisting of 2922 highly expressed genes in the TK and 2349 highly expressed genes in the spleen (Fig. 3B).

The genes that exhibited higher expression in one organ compared to others were considered organ-dependent DEGs. We analyzed the tissue-dependent DEGs in each organ based on the DEGs between any two organs. 1529, 1368, and 47 organ-dependent DEGs were identified in the spleen, TK and HK, respectively. The organ-dependent DEGs in HK was the least.

### Functional characterization of organ-dependent DEGs

**Fig. 4:**
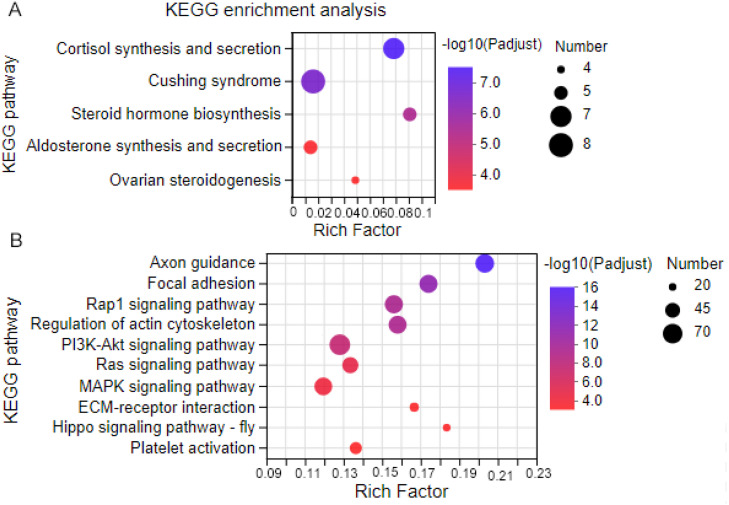
The enriched KEGG pathways of organ-dependent DEGs. (A) The enriched pathways of organ-dependent DEGs in the head kidney. (B). The enriched pathways of organ-dependent DEGs in the spleen (Top 10). The X-ray represents the rich factor. The Y-ray represents an enriched pathway. "Number" means the number of DEGs enriched in the KEGG pathway. "Padjust" represents the false discovery rate.

The KEGG and GO enrichment analyses were conducted using the tissue-dependent DEGs to assess the organ functions. In the HK, GO analysis revealed the 47 organ-dependent DEGs were categorized into 126 GO terms. The GO enrichment analysis showed three GO terms (GO:0006694, GO:0008202, GO:0008207) were statistically enriched (corrected p-value < 0.05, the same below). The KEGG pathway enrichment analysis was conducted to better understand organ-dependent DEGs functions. The KEGG pathway analysis showed five pathways were statistically enriched, including the cortisol synthesis and secretion pathway, steroid hormone biosynthesis pathway, aldosterone synthesis and secretion, and so on (Fig. 4A).

In the spleen, 1529 organ-dependent DEGs were categorized into 317 GO terms and 293 KEGG pathways. 68 GO terms were statistically enriched. The cellular anatomical entity term (GO:0110165) was enriched with the highest number of DEGs, followed by the intrinsic component of the membrane term. Based on the analysis results, there were 27 significantly enriched KEGG pathways, and the enriched pathway with the highest number of DEGs was PI3K-Akt signaling pathway. Besides, some organ-dependent DEGs were enriched in immune-related pathways, including hematopoietic cell lineage, platelet activation, natural killer cell-mediated cytotoxicity, and Fc gamma R-mediated phagocytosis pathway (Fig. 4B).

For the 1368 organ-dependent DEGs in the TK, a total of 359 GO terms and 297 KEGG pathways were enriched. Based on the results, 105 GO terms and 36 KEGG pathways were statistically enriched. The enriched GO terms were related to several catabolic processes, microtubule-based movement, and other GO terms. In addition, enriched KEGG pathways were related to several metabolic pathways, including mineral absorption, peroxisome, and others. Interestingly, the DEGs associated with steroid hormone biosynthesis were also observed in the TK (Supplementary Fig. 6).

### The expression features of immune-related genes

**Fig. 5:**
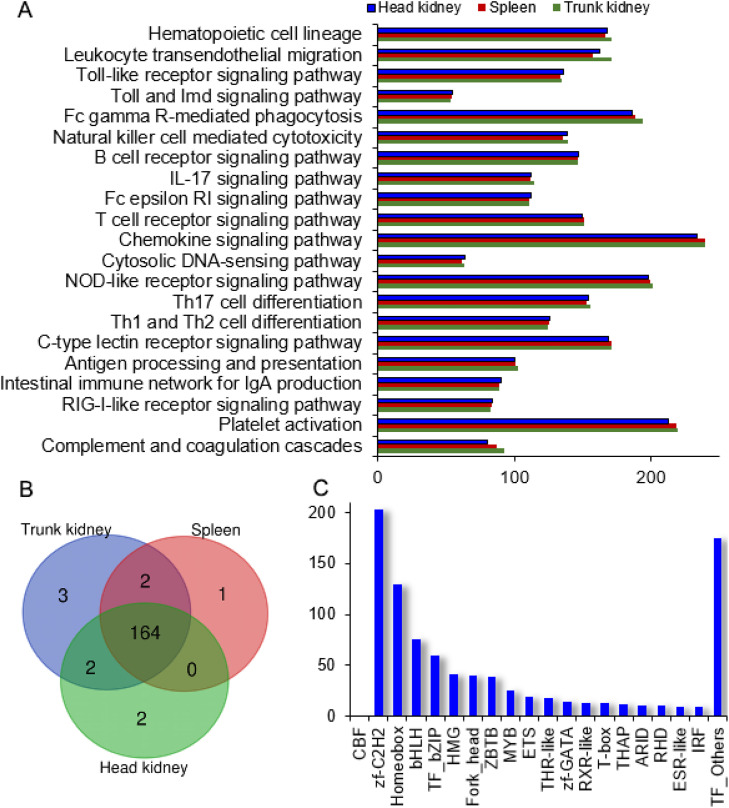
The analysis of TF, immune-related and hematopoietic gene. (A) The expressed genes involved in the immune system (KEGG second category). The x-axis represents the number of genes, and the y-axis represents pathways. (B) The Venn diagram of hematopoietic related genes in three organs. (C) Top 20 TF families.

According to the results of KEGG databases analysis, 1407 genes involved in immune-related pathways were annotated, with 1368, 1313 and 1328 genes expressed in TK, HK and spleen, correspondingly (Fig. 5A). In total, 415 DEGs were identified between HK and spleen, of which 113 genes were highly expressed in HK and 302 were higher in spleen. The expressed levels of 151 genes were higher in TK and 266 genes were higher in spleen. Except *egr3-like*, 82 genes were highly expressed in TK vs. HK. The expression levels of some immune-related genes were similar in the three organs, with some variations, including complement *c2, c4, il8*/*34, c-x-c13, c-c19, nod1, nod2*, and so on. Some immune-related genes were highly expressed in TK and HK, such as cf*h, il17a, ncf2/4, tlr5, ccl3, c*6, *c7*, and *il10*. Some genes were highly expressed in the spleen and TK, such as f3, *tnfsf10*. Other immune-related genes highly expressed in the spleen, included *tnf, tnfsf6, il17f, c-x-c10/14*, tlr*1*/*3*/*7*/*8, scarb1, pfp1, gp1bb, CD3e*, CD3z and others. Additionally, the higher expression levels of *cfd* and *f3* were observed in the TK (Tables 2, S2).

**Table 2:** The differentially expressed genes among the head kidney, trunk kidney, and spleen.

Gene description	Gene name	Expression level in spleen	Expression level in HK	Expression level in TK	Log_2_(HK/S)	Log_2_(TK/S)	Log_2_(TK/HK)
Tumor necrosis factor ligand superfamily member 10	*Tnfsf10*	6.19	1.14	10.62	−1.94	1.09	3.03
Complement C6	*C6*	11.00	28.09	24.47	1.88	1.47	−0.39
Complement C7	*C7*	1.91	3.37	5.55	1.29	1.83	0.54
Interleukin-17a	*Il17a*	0.26	2.71	2.06	4.06	3.54	−0.51
Cd3e molecule	*Cd3e*	28.64	10.45	11.46	NO	−1.02	−0.03
Neutrophil cytosolic factor 4	*Ncf4*	11.95	36.77	30.36	2.11	1.66	−0.44
Toll-like receptor 3	*Tlr3*	10.7	1.64	3.18	−2.19	−1.43	0.78
Toll-like receptor 5	*Tlr5*	0.38	2.64	2.17	3.25	2.81	−0.42
Toll-like receptor 7	*Tlr7*	4.67	1.39	1.29	−1.25	−1.56	−0.31
Toll-like receptor 8	*Tlr8*	16.05	5.38	6.11	−1.09	−1.09	−0.006
Macrophage receptor with collagenous structure	*Marco*	656.88	173.12	275.31	−1.39	−0.92	0.47
Colony stimulating factor 3 receptor	*Csf3r*	33.88	131.54	111.51	2.46	2.03	−0.42
CD44 molecule	*Cd44*	25.91	58.62	55.03	1.66	1.39	−0.26
Erythropoietin	*Epo*	2.05	0.11	0.31	−3.80	−2.43	N
Kit ligand	*Kitlg*	2.63	0.65	0.64	−1.47	−1.72	−0.23
Macrophage colony-stimulating factor 1 receptor 1	*Csf1r*	67.93	11.44	20.5	−2.09	−1.42	0.67

### The expression features of hematopoietic related genes

To better characterize hematopoiesis function in three organs, the genes related to hematopoietic cell lineage pathway were analyzed. The sequencing data showed that 175 genes were involved in hematopoietic cell lineage pathway, with 171, 168 and 167 genes expressed in TK, HK and spleen, correspondingly. A total of 164 genes were expressed in three organs (Fig. 5B). The DEGs between any two organs were analyzed. The expression levels of some genes were similar in three organs, including *cd4, il7r, cd86, tnfrsf5, cd68, crsg, epor, csf1r2* and so on.

Notably, some genes were differentially expressed in the three organs. Except for *kita*, 8 DEGs were identified between TK and HK, with highly elevated expression in TK. A total of 50 genes were differentially expressed, including 16 genes in HK and 34 genes in spleen. In total, 68 DEGs were identified, of which 18 genes were highly expressed in TK and 60 genes were highly expressed in spleen. Several essential genes were highly expressed in both HK and TK, including *csf3r, cd44, mme*. However, some genes were highly expressed in the spleen, such as *kitlg, cd34, csf1r1* and *epo* (Table 2).

### Transcription factor analysis

According to reports, the transcription factor (TF) network plays critical roles in hematopoietic cell differentiation. A total of 923 TFs were identified, which belonged to 66 different TF families. The top three TF families were Cys2-His2 zinc finger (Zf-C2H2), Homeobox and basic helix-loop-helix (bHLH) (Fig. 5C). Of these TFs, 782 genes were expressed in three organs. Some TFs were exclusively expressed in specific organs: 15 in HK, 46 in TK and 26 in spleen. Transcription factors (TFs) play pivotal roles in haematopoietic lineage commitment. To elucidate the hematopoietic function of three organs, the expression patterns of some key TFs were analyzed. The TFs involved in hematopoiesis, such as *tal1, runx1, myb, cbfβ, cebpa, meis1, gata2, gata3, pu.1, pax5*, were expressed in three organs. The expressed levels of these TFs were not significantly different between HK and TK. Some TFs, such as *cmyb* and *cebpa,* were highly expressed in HK and TK.

### Proposed model of definitive hematopoietic differentiation

**Fig. 6:**
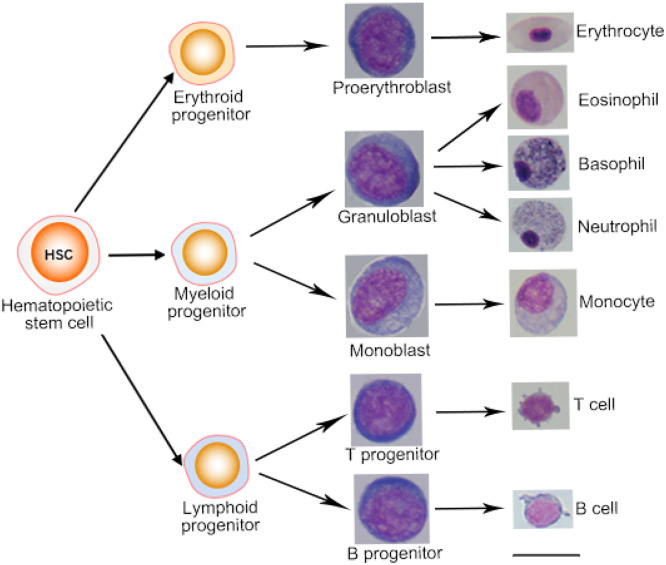
The differentiation progress of definitive hematopoiesis.

Wright's-Giemsa stained blood smears and tissue imprints were observed under a microscope at 10 × 100 magnification and different blood cells were determined according to cell morphology, cell size, nuclear size, and color of intracellular granule. The proerythroblast had a large nucleus and coarser nuclear chromatin. A small pale area was often observed in the rim of the basophilic cytoplasm. The granuloblast was similar to the erythroblast but had a bigger size and more cytoplasm. The monoblast was the biggest blast cell with abundant blue cytoplasm. The T and B progenitors were smaller than other blast cells (Fig. 6). The mature blood cells included the oval-shaped erythrocytes, eosinophils with dark-red granules in cytoplasm, basophils with dark purple large granules, neutrophils with blue-purple and reddish small granules, monocytes with the biggest cell size in blood smears, T cells and B cells. The nucleus of T and B cell occupied most of the cell volume (Fig. 6).

The hematopoietic tree was established using cell surface markers and morphologic features. The mature hematopoietic stem cells differentiated into progenitors, including the myeloid progenitors with Csf3r (Cd114), erythroid progenitors with Epor, and lymphoid progenitor cells with Il7r. Subsequently, myeloid progenitors given rise to myeloblast and monoblast, which underwent several recognizable morphologic stages and ultimately developed into neutrophils, eosinophils, basophils, and monocytes. The lymphoid progenitor cells given rise to T and B progenitors, which differentiated into the T and B cells. The conventional T-cell population comprised T-helper cells, regulatory T cells, and cytotoxic T cells (Fig. 6). Natural killer cell surface marker Cd56 was detected in three organs.

### Confirmation of some immune-related DEGs expression

Four immune-related genes were selected for real-time PCR to validate the mRNA sequencing results. *C7, tlr5*, and *csf3r* showed high expression levels in HK and TK, while the expressed abundance of *itga3* was not significant in any of the three organs (Supplementary Fig. 7). The qPCR and RNA-Seq data showed similar expression profiles.

## Discussion

### The haematopoietic function of three organs

The haematopoietic organs in teleosts are distinct from those operating in mammalian because they lack bone marrow [13]. Instead of bone marrow, teleosts use the HK, TK, and spleen as the site for hemopoiesis. However, the gene expression profiles of these organs were poorly reported. This study comprehensively analyzed the sections, imprints, and transcriptomes of HK, TK, and spleen from adult yellow large croaker.

Hematopoiesis is an evolutionarily conserved process among vertebrates [51]. Studies in zebrafish and mouse demonstrate that Runx1 is required for the generation and formation of hematopoietic stem cells (HSCs) during the definitive hematopoiesis [51,52]. *Cmyb* is primarily expressed in HSCs and progenitor cells in human and mouse [53,54]; c*myb* mutation results in failure of definitive hematopoiesis in zebrafish and medaka, indicating *cmyb* is necessary for formation of HSCs and HSPCs [55,56]. Recent research has revealed that CD133 (PROM1) is a surface marker of mature hematopoietic stem cells (HSCs) in humans, while the gene characteristic for progenitors includes cytokine receptors CSF3R associated with myeloid progenitors, IL7R and IL2RG associated with lymphoid progenitors and EPOR associated with erythroid progenitors [57]. In zebrafish, granulocyte colony-stimulating factor (Gcsf or Csf3) is a cytokine responsible for the differentiation and proliferation of monocytes, macrophages, granulocytes and their respective progenitors by binding to its receptor, Gcsfr (Csf3r) [58]. *Runx1* and *cmyb* were expressed in three organ of large yellow croaker. The other key genes involved in hematopoietic differentiation were also found in three organs, such as *cd133, kit, kit ligand, cd10, cd34, cd135, epor, csf3r* and *il7r*. In addition, the essential hematopoietic transcription factors were observed in three organs, including *tal1, cbfβ, meis1, gata2, pu.1, gata3, hoxa5*, and others. The comprehensive transcriptomic profiling results indicated that these three organs have the hematopoietic function.

HK is considered the primary hemopoietic organ of teleosts based on cellular composition, responsible for the maturation of erythrocyte, B cells, monocyte, granulocytes, and generation of T-cell progenitors [[59], [60], [61], [62]]. In this study, we found the expression levels of *runx1* and *cmyb* were similar in HK and TK. The expression of other key genes involved in hematopoiesis, such as *epor, csf3r, il7r, tal1, cbfβ, meis1, gata2, pu.1, gata3*, were not significantly different between HK and TK. The hematopoietic tissues were also observed in HK and TK. Thus, the results indicated that both HK and TK were the primary hemopoietic organs of large yellow croaker and were the important sites for the production of HSPCs, erythrocyte, B cells, monocyte and granulocytes. Interestingly, *cmyb* and *csf3r* were highly expressed in HK and TK, indicating that more HSPCs and myeloid cells housed in HK and TK.

The hemopoietic function of spleen has been reported in a few fish, because spleen is generally considered as a major lymphoid organ [[11], [12], [13],^21^,^27^]. In this study, *runx1* and *cmyb* were expressed in spleen. Furthermore, the immature blood cells were observed in large yellow croaker spleen. These results indicated the presence of HSPCs or immature progenitors in spleen, consistent with results in zebrafish that HSPCs have been found in zebrafish spleen by single-cell transcriptome sequencing [32]. However, it remains unclear whether HSPCs or immature progenitors differentiate into mature blood cells in spleen.

### The immune function of three organs

In teleosts, macrophages play an essential role in presenting antigens by separating and concentrating the phagocytosed material [63]. The macrophage surface molecule *Marco* has been detected in the HK, TK and spleen of the large yellow croaker [64,65]. The melano-macrophages, an leukocyte populations different from higher vertebrates, can be loosely dispersed or integrated to form MMCs that are aggregates of dark, pigmented phagocytes within the immune organs [28]. These cells play roles in various infections, such as bacterial and viral diseases [28,[66], [67], [68], [69]]. The MMCs in fish have mainly been found in HK and spleen [28,[66], [67], [68], [69]]. In this study, the sections of HK, TK and spleen were observed. The results showed the presence of MMCs in all three organs, indicating TK were also the main immune organs.

The organized secondary lymphoid microstructures (SLMs), such as germinal centers (GCs), facilitate the encounter between antigen, antigen-presenting cells, and T/B lymphocytes, and promote the B cell clonal expansion and maturation that results in high-affinity antibody responses [[69], [70], [71]]. It is thought that the organized SLMs first arose in birds, but recent study confirmed germinal center-like structures in cold-blooded vertebrates [69]. These structures, termed M-Las, are inducible microstructures composed of MMCs and B and T cell aggregates, in which B cells undergo proliferation, clonal expansion and somatic hypermutation processes [69]. The marker genes of B and T cells were annotated in three organs, such as *cd79a, cd22, cd4, cd3e* and *cd3z*. The MMCs and associated lymphocyte aggregates were observed in three organs in large yellow croaker, indicating that these organized structures may function similarly to mammalian GCs. Further validation is needed, but the results support that HK and TK function as the secondary lymphoid organs in large yellow croaker.

The Toll-like receptor (TLR) family are important pattern-recognition receptors [72]. TLRs recognize multiple endogenous and exogenous ligands. TLR3 recognizes double-stranded RNA and activates interferon response genes in virus dsRNA-induced innate immune response [73]. TLR7 and TLR8 are endosomal receptors that play a crucial role in response to viral infection by recognizing GU-rich short single-stranded RNA (ssRNA) [74]. Notably, *tlr3, tlr7*, and *tlr8* were highly expressed in the spleen, suggesting that the spleen plays an essential role in the antiviral immune response, including the production and secretion of type I interferon (IFN) and pro-inflammatory cytokines.

The complement system comprises approximately 20 distinct serum proteins that serve various functions in the innate and adaptive immune system, including microbial killing, inflammatory reactions, phagocytosis, and antibody production [75]. *C3, c4, c6*, and *c7* were observed in three organs of large yellow croaker, indicating that these organs are also involved in secretion. C8 is a vital component of the cytolytic membrane attack complex and comprises three subunits: C8α, C8β, and C8γ [76]. Interestingly, in large yellow croaker, *c8γ* was only observed in TK. A similar phenomenon has been observed in yellow catfish [35]. Therefore, we had reason to believe that the TK existed the special C8γ-synthesizing cells.

### Connections and interactions between hematopoiesis and immune function

The HK, TK and spleen of large yellow croaker integrate hematopoietic and immune functions, maybe reflect an evolutionary strategy to optimize physiological efficiency in aquatic environments. Innate immune cells, such as macrophages and neutrophils, originate from hematopoietic stem/progenitor cells (HSPCs) in the head kidney, while these cells secrete several mediators (e.g., IL-1β, IL-6, TNF-α) that regulate hematopoiesis, creating a loop essential for maintaining homeostasis [[77], [78], [79]]. This crosstalk ensures rapid replenishment of immune cells during stress while preventing excessive inflammation—a balance critical for survival in pathogen-rich aquatic habitats. The structural organization of these organs facilitates this integration. Stromal cells and extracellular matrix components form specialized microenvironments that support both hematopoietic differentiation and immune cell maturation. The spatial coordination allows immune cells to directly interact with hematopoietic precursors, enabling real-time modulation of cell production based on immunological needs. The presence of hematopoietic and immune tissues in same organ may represent a foundational aspect of their physiology, essential for sustaining health and resilience in varying environments.

## Conclusion

In teleosts, the immune function of TK and haematopoietic function of spleen are rarely reported. In this study, the smears, sections and transcriptome of HK, TK and spleen were analyzed. The hematopoiesis-related genes, including *cd133, tal1, runx1, kit, kit ligand, myb, epor, il7r, il2rg* and *csf3r*, were identified in three organs. The surface markers of B and T cells were observed in three organs, including *cd*ε, *cd*3ζ, *cd*79α, and so on. Both HK and TK were highlighted as primary hematopoietic organs, while the spleen also exhibited hematopoietic activity. The organized MMCs and lymphocyte aggregates were observed in three organs, indicating their roles as secondary lymphoid organs. Many immune-related genes were highly expressed in spleen, including *tlr3, tlr7* and *tlr8*. Complement *c6* and *c7* were highly expressed in HK and TK. *C8γ* (TPM:13.42) was only observed in TK. The results prompt further validation of the immune and hematopoietic functions using techniques like CRISPR-mediated knockout, morpholino-mediated knockdown, and knock-in studies. The results are beneficial to comprehending the molecular mechanisms and contribute to disease management in large yellow croaker. Such studies in large yellow croaker may also help to understand the immunity and regulatory mechanism in teleost.

## Ethics statement

The experiment process was conducted according to the Committee on Laboratory Animal Welfare and Ethics of Zhejiang Ocean University.

## CRediT authorship contribution statement

**Aihua Zhong:** Writing – original draft. **Yingbin Wang:** Resources. **Haiqi Zhang:** Writing – review & editing. **Xiaojun Yan:** Project administration, Funding acquisition, Conceptualization. **Shuo Jia:** Writing – review & editing.

## Declaration of competing interest

The authors declare that they have no known competing financial interests or personal relationships that could have appeared to influence the work reported in this paper.

## Data Availability

Data will be made available on request.

## Appendix Supplementary materials

Image, application 1
