# Supplementary material for: Histological and transcriptome analysis provides new insights into the hematopoietic and immune functions of head kidney, trunk kidney and spleen of adult large yellow croaker, Larimichthys crocea
Source: Comp Immunol Rep. 2025 Apr 12;8:200223. doi: 10.1016/j.cirep.2025.200223 (PMC12020876; doi:10.1016/j.cirep.2025.200223)
Supplement: Supplementary file 1 [file mmc1.docx]

Supplement


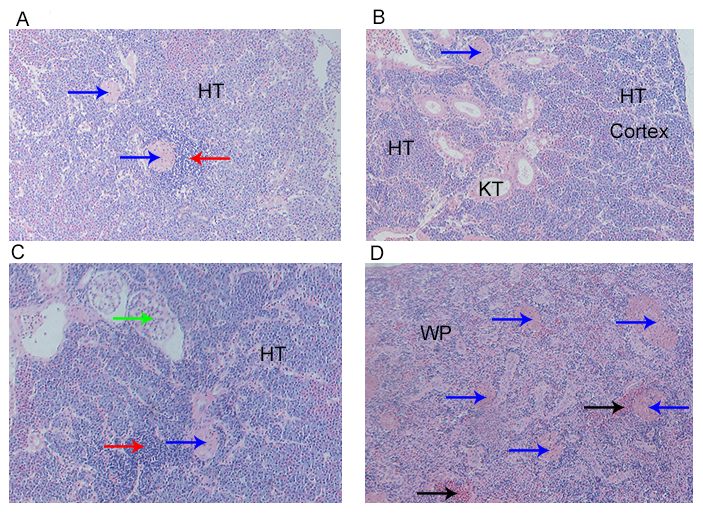


Fig.1. The section from the large yellow croaker. (A) The section from the head kidney (10×10). (B-C) The section from the trunk kidney (10×10). (D) The section from the spleen (10×10). Hematopoietic tissue (HT). Kidney tubule (KT). White pulp (WP). Blue arrow: Melanomacrophage aggregate. Red arrow: lymphoid zone. Black arrow: red pulp. Green arrow: renal capsule.


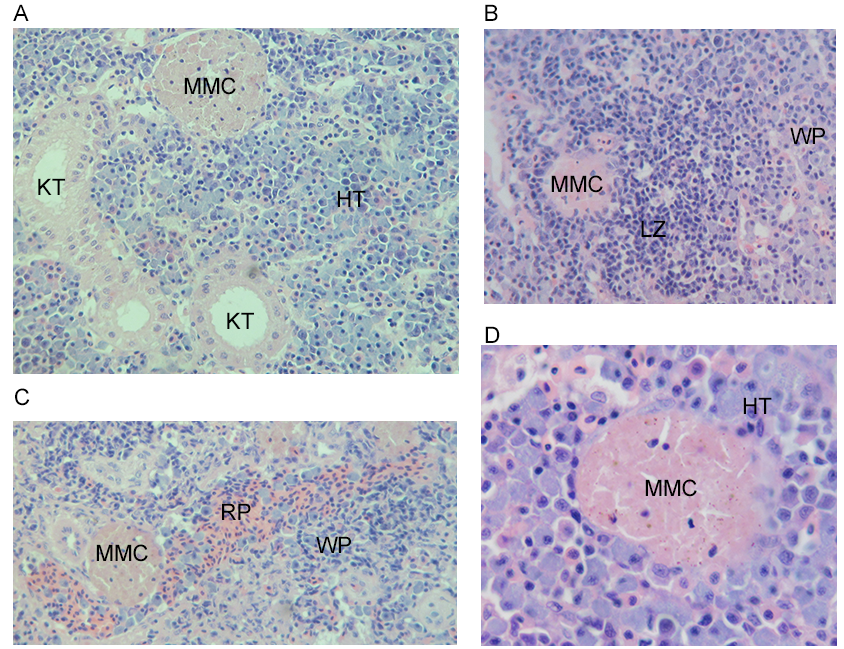


Fig.2. Sections from the large yellow croaker. (A) The section from the trunk kidney (10×40). (B) The section from the spleen (10×40). (C) The red pulp in spleen (10×40). (D) The pigmented materials in Melanomacrophage aggregate or MMC (10×100). Melanomacrophage aggregate (MMC). Kidney tubule (KT). Hematopoietic tissue (HT). Lymphoid zone (LZ). Red pulp (RP). White pulp (WP).


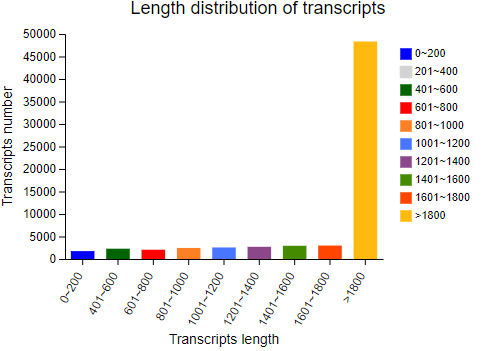


Fig.3. Transcript length distribution. The x-axis represents the transcript length, and the y-axis represents the transcript number.


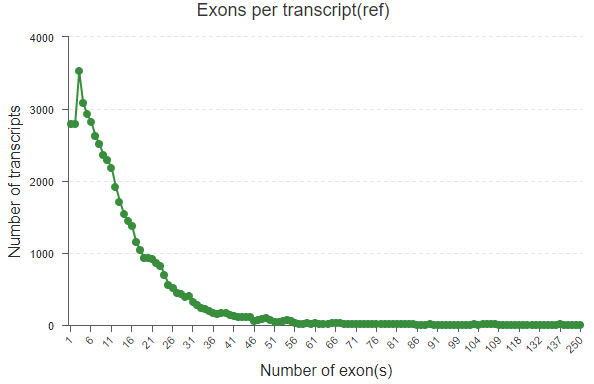


Fig. 4. The relationships between the numbers of transcripts and exons. The x-axis represents the numbers of exons, and the y-axis represents the transcript number.


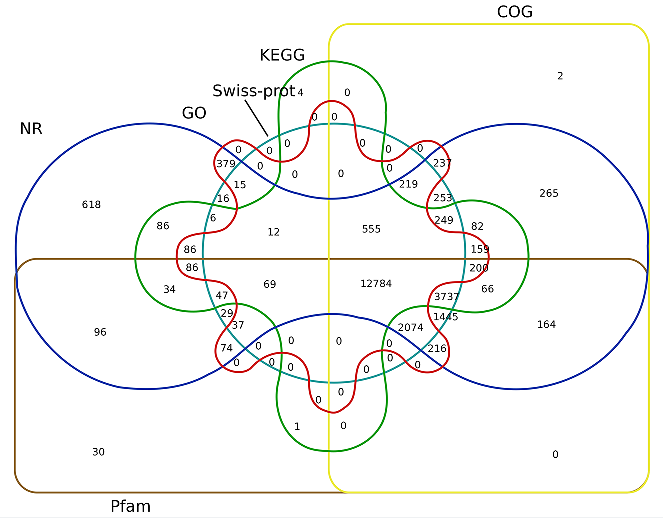


Fig. 5. Venn diagram of unigenes annotated in six databases


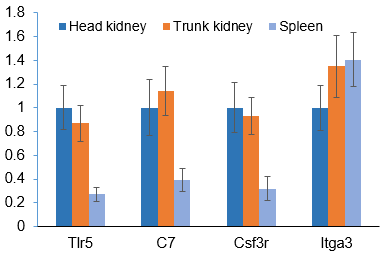


Fig. 6. The quantitative real-time PCR validation of immune related genes in three organs

**Table S1 Statistical evaluation of sequencing data**

| Sample | Raw reads | Raw bases | Clean reads | Clean bases | Q20(%) | Q30(%) | GC content(%) |
| --- | --- | --- | --- | --- | --- | --- | --- |
| Spleen1 | 54,552,674 | 8,237,453,774 | 53,700,072 | 7,893,340,849 | 97.67 | 93.43 | 50.78 |
| spleen2 | 53,229,734 | 8,037,689,834 | 52,658,008 | 7,705,695,424 | 98.36 | 95.13 | 50.78 |
| spleen1 | 55,210,138 | 8,336,730,838 | 54,573,298 | 8,006,110,944 | 98 | 94.21 | 50.54 |
| TK1 | 64,483,998 | 9,737,083,698 | 62,996,274 | 8,854,075,994 | 98.01 | 94.32 | 50.75 |
| TK2 | 54,817,698 | 8,277,472,398 | 53,956,336 | 7,821,048,324 | 98.03 | 94.27 | 50.97 |
| TK1 | 46,886,722 | 7,079,895,022 | 46,422,226 | 6,834,964,088 | 98.24 | 94.77 | 50.87 |
| HK1 | 56,477,080 | 8,528,039,080 | 55,663,614 | 8,073,265,533 | 98 | 94.21 | 51.06 |
| HK2 | 49,618,904 | 7,492,454,504 | 48,712,162 | 7,081,366,649 | 97.63 | 93.35 | 50.97 |
| HK3 | 51,030,972 | 7,705,676,772 | 50,360,426 | 7,321,386,095 | 98.3 | 94.92 | 51.18 |

TK: trunk kidney, HK: head kidney

**Table S2 Differentially expressed genes in the head kidney, trunk kidney, and spleen**

| Gene description | Gene name | Expression level in spleen | Expression level in HK | Expression level in TK | | Log_2_(HK/S) | | Log_2_(TK/S) | | Log_2_(TK/HK) | |
| --- | --- | --- | --- | --- | --- | --- | --- | --- | --- | --- | --- |
| Tumor necrosis factor ligand superfamily member 10 | *Tnfsf10* | 6.19 | 1.14 | 10.62 | | -1.94 | | 1.09 | | 3.03 | |
| Complement factor H | *Cfh* | 18.4 | 46.78 | 44.57 | | 1.76 | | 1.49 | | -0.25 | |
| Nuclear factor of activated t-cells, cytoplasmic 1 | *Nfatc1* | 2.12 | 0.49 | 0.45 | | -1.62 | | -1.92 | | -0.31 | |
| Tumor necrosis factor | *Tnf* | 6.79 | 1.13 | 1.44 | | -2.11 | | -1.95 | | 0.16 | |
| Tumor necrosis factor ligand superfamily member 6 | *Tnfsf6* | 20.92 | 5.74 | 6.05 | | -1.32 | | -1.42 | | -0.09 | |
| Interleukin-17a | *Il17a* | 0.26 | 2.71 | 2.06 | | 4.06 | | 3.54 | | -0.51 | |
| Interleukin-17f | *Il17f* | 36.59 | 1.19 | 1.91 | | -4.39 | | -3.89 | | 0.51 | |
| C-X-C motif chemokine 10 | *Cxcl10* | 192.61 | 50.78 | 69.33 | | -1.43 | | -1.11 | | 0.33 | |
| Matrix metallopeptidase 9 | *Mmp9* | 79.83 | 285.29 | 223.78 | | 2.35 | | 1.79 | | -0.54 | |
| Matrix metalloproteinase-13 (collagenase 3) | *Mmp13* | 18.13 | 117.83 | 100.15 | | 3.06 | | 2.73 | | -0.33 | |
| Interleukin-6 receptor subunit alpha | *Il6ra* | 14.67 | 37.93 | 34.06 | | 1.91 | | 1.56 | | -0.34 | |
| Epstein-Barr virus induced 3 | *Ebi3*, *il27b* | 3.72 | 6.15 | 5.92 | | 1.23 | | 1.02 | | 0.63 | |
| Interleukin 2 receptor subunit beta | *Il2rb* | 49.53 | 9.91 | 15.17 | | -1.68 | | -1.47 | | 0.22 | |
| Interleukin-12 receptor subunit beta-2 | *Il12rb2* | 27.65 | 6.92 | 7.58 | | -1.51 | | -1.55 | | -0.03 | |
| Cd3e molecule | *Cd3e* | 28.64 | 10.45 | 11.46 | | NO | | -1.02 | | -0.03 | |
| T-cell surface glycoprotein CD3 zeta chain | *Cd3z* | 31.91 | 4.98 | 6.77 | | -2.20 | | -1.90 | | 0.31 | |
| Toll-like receptor 8 | *Tlr8* | 16.05 | 5.38 | 6.11 | | -1.09 | | -1.09 | | -0.006 | |
| Neutrophil cytosolic factor 4 | *Ncf4* | 11.95 | 36.77 | 30.36 | | 2.11 | | 1.66 | | -0.44 | |
| Neutrophil cytosolic factor 2 | *Ncf2* | 33.13 | 59.57 | 54.03 | | 1.34 | | 1.03 | | -0.31 | |
| Class II major histocompatibility complex transactivator | *Ciita* | 27.71 | 9.99 | 10.32 | | -1.02 | | -1.13 | | -0.13 | |
| Complement C6 | *C6* | 11.00 | 28.09 | 24.47 | | 1.88 | | 1.47 | | -0.39 | |
| Complement C7 | *C7* | 1.91 | 3.37 | 5.55 | | 1.29 | | 1.83 | | 0.54 | |
| Interleukin 10 | *Il10* | 0.74 | 1.32 | 5.38 | | 1.32 | | 3.25 | | 1.93 | |
| Interleukin-12 subunit beta | *Il12b* | 7.58 | 0.62 | 0.61 | | -3.12 | | -3.33 | | -0.21 | |
| C-X-C motif chemokine 10 | *Cxc10* | 192.61 | 50.78 | 69.33 | | -1.42 | | -1.11 | | 0.33 | |
| Toll-like receptor 1 | *Tlr1* | 49.16 | 15.78 | 17.82 | | -1.25 | | -1.28 | | -0.02 | |
| Toll-like receptor 3 | *Tlr3* | 10.7 | 1.64 | 3.18 | | -2.19 | | -1.43 | | 0.78 | |
| Toll-like receptor 7 | *Tlr7* | 4.67 | 1.39 | 1.29 | | -1.25 | | -1.56 | | -0.31 | |
| Nuclear factor of activated T cells 4 | *Nfatc4* | 3.73 | 0.61 | 0.65 | | -2.14 | | -2.23 | | -0.08 | |
| C-X-C chemokine receptor type 4 | *Cxcr4* | 109.34 | 345.93 | 306.31 | | 2.16 | | 1.82 | | -0.33 | |
| Complement factor D | *Cfd* | 140.71 | 61.02 | 328.72 | | -0.67 | | 1.57 | | 2.25 | |
| Coagulation factor III | *F3* | 17.41 | 5.69 | 31.51 | | -1.39 | | 1.04 | | 2.44 | |
| Macrophage receptor with collagenous structure | *Marco* | 656.88 | 173.12 | 275.31 | | -1.39 | | -0.92 | | 0.47 | |
| Scavenger receptor class B member 1 | *Scarb1* | 40.06 | 2.03 | 2.21 | | -3.75 | | -3.84 | | -0.08 | |
| Perforin-1 | *Prf1* | 100.79 | 3.27 | 4.59 | | -4.47 | | -4.14 | | 0.33 | |
| Perforin-1, transcript variant X4 | *Prf1* | 45.15 | 1.91 | 2.07 | | -3.61 | | -3.24 | | 0.36 | |
| C-X-C motif chemokine ligand 14 | *Cxcl14* | 77.86 | 1.03 | 0.32 | | -5.63 | | -7.66 | | -2.01 | |
| Monocyte chemotactic protein 1B | *Ccl2*, *mcp1* | 143.14 | 26.77 | 34.22 | | -1.89 | | -1.59 | | 0.32 | |
| C-C motif chemokine 3 | *Ccl3* | 40.18 | 11.56 | 11.36 | | -1.33 | | -1.45 | | -0.11 | |
| C-X-C chemokine receptor type 2 | *Cxcr2*, *cd182* | 10.11 | 80.43 | 70.88 | | 3.49 | | 3.15 | | -0.33 | |
| C-X-C chemokine receptor type 4 | *Cxcr4*, *cd184* | 109.34 | 345.93 | 306.31 | | 2.15 | | 1.81 | | -0.33 | |
| Alkaline phosphatase | *Alpl* | 2.39 | 61.42 | 79.82 | | 5.17 | | 5.36 | | 0.19 | |
| Toll-like receptor 5 | *Tlr5* | 0.38 | 2.64 | 2.17 | | 3.25 | | 2.81 | | -0.42 | |
| Prominin-1-A | *Prom1cd133* | 10.63 | 35.35 | 28.81 | 2.21 | | 1.72 | | -0.47 | |  |
| Fms related tyrosine kinase 3 | *Flt3*, *cd135* | 439.05 | 23.44 | 27.96 | -3.76 | | -3.65 | | 0.12 | |  |
| Kinase inserts domain receptor | *Kdr*, *vegfr2*, *cd309* | 56.87 | 4.88 | 8.49 | -3.05 | | -2.42 | | 0.63 | |  |
| Tumor necrosis factor receptor superfamily member 16 | *Ngfr*,*tnfrsf16*, *cd271* | 17.94 | 2.01 | 1.76 | -2.76 | | -3.12 | | -0.35 | |  |
| ADP-ribosyl cyclase/cyclic ADP-ribose hydrolase 1 | *Cd38* | 14.31 | 4.39 | 14.02 | -1.23 | | 0.27 | | 1.51 | |  |
| MYB proto-oncogene | *Myb* | 4.62 | 37.37 | 35.11 | 3.55 | | 3.28 | | -0.27 | |  |
| CD34 molecule | *Cd34* | 0.80 | 7.18 | 6.83 | 3.69 | | 3.45 | | -0.23 | |  |
| CCAAT/enhancer-binding protein beta | *Cebpb* | 136.97 | 627.75 | 573.02 | 2.69 | | 2.39 | | -0.29 | |  |
| Colony stimulating factor 3 receptor | *Csf3r*, *cd114* | 33.88 | 131.54 | 111.51 | 2.46 | | 2.03 | | -0.42 | |  |
| CD44 molecule | *Cd44* | 25.91 | 58.62 | 55.03 | 1.66 | | 1.39 | | -0.26 | |  |
| Membrane metalloendopeptidase | *Mme*, *cd10* | 14.03 | 73.16 | 70.02 | 2.91 | | 2.67 | | -0.23 | |  |
| Interleukin-6 receptor subunit alpha | *Il6r*, *cd126* | 14.66 | 37.92 | 34.06 | 1.91 | | 1.56 | | -0.34 | |  |
| Erythropoietin | *Epo* | 2.05 | 0.11 | 0.31 | -3.80 | | -2.43 | | N | |  |
| KIT ligand | *Kitlg* | 2.63 | 0.65 | 0.64 | -1.47 | | -1.72 | | -0.23 | |  |
| Macrophage colony-stimulating factor 1 receptor 1 | *Csf1r*, *cd115* | 67.93 | 11.44 | 20.5 | -2.09 | | -1.42 | | 0.67 | |  |
